# Inhibitory Control Training for Anxiety and Math Achievement in Primary School Children: Protocol for a Proof-of-Concept Study

**DOI:** 10.2196/52929

**Published:** 2024-03-13

**Authors:** Elizabeth Edwards, Khanh Linh Chu, Annemaree Carroll

**Affiliations:** 1 The University of Queensland St Lucia Australia

**Keywords:** cognitive control training, anxiety, inhibitory control, math achievement

## Abstract

**Background:**

Cognitive control training (CCT) has shown potential to reduce emotional vulnerability in adults and adolescents. However, there is scant literature testing the efficacy of CCT for the reduction of anxiety and transferring the effects to educational outcomes in children. Building on the evidence that a greater ability to suppress a prepotent response (inhibitory control) is associated with higher math achievement in children, it is plausible that training inhibitory processes using a CCT paradigm may be beneficial for reducing anxiety, improving inhibitory control, and in turn increasing math achievement.

**Objective:**

This proof-of-concept study aims to investigate the efficacy of 15 sessions of inhibitory control training for reduction in anxiety and improvement in math achievement in primary school children.

**Methods:**

We will use a 2 (group: CCT, adaptive Go/No-Go vs active control, low-load task) multiplied by 4 (time: pre- vs posttraining vs 1-month vs 3-month follow-up) randomized design in a nonselected sample of 100 children aged 8-10 years. Both groups will complete 10 minutes of daily training for 3 weeks at school. The dependent variables will be anxiety and correlates (Spence Children’s Anxiety Scale, Penn State Worry Questionnaire for Children, Revised Children’s Anxiety and Depression Scale, Child Response Style Questionnaire, and Modified Abbreviated Math Anxiety Scale), inhibitory control (Go/No-Go task), shifting (color-shape shifting task), updating (*n*-back task), and math achievement (Applied Problems, Calculation, and Math Facts Fluency subtests from the Woodcock-Johnson IV Tests of Achievement).

**Results:**

We opened enrollment in September 2023. The initial results are expected to be published in late 2024. We predict that children in the CCT group will show a reduction in emotional symptoms; improvements in inhibition, shifting, and updating performance; and advances in math achievement from pre- to posttraining, and that these effects will be maintained at 1- and 3-month follow-ups, compared to children in the active control group.

**Conclusions:**

The CCT paradigm used in our study will provide a greater understanding of the emotional and cognitive transfer effects on children and inform future work. Specifically, the findings will advance the knowledge of deploying inhibitory control training with children and provide valuable insights into its use for reducing anxiety and advancing math achievement.

**Trial Registration:**

Open Science Framework ofs.io/de2qa; https://doi.org/10.17605/OSF.IO/DE2QA

**International Registered Report Identifier (IRRID):**

PRR1-10.2196/52929

## Introduction

### Overview

Anxiety is the most reported mental health problem in young people across and beyond the pandemic [1,2]. Childhood anxiety is associated with excessive worry, avoidance, physical symptoms, and severe social and academic problems [3]. Interventions for anxiety in children are typically expensive to administer (eg, 1:1 psychological therapy) with long waitlists to see providers in the public health system. Hence, there is a need for novel approaches to closing the treatment gap, that is, reducing the discrepancy between the percentage of children with mental health problems and the percentage who receive treatment [4]. The focus of this proof-of-concept study is treating childhood anxiety by targeting the modifiable cognitive processes that underpin emotional symptoms. We will deliver an intervention at school as part of regular classroom routines and examine its efficacy in reducing anxiety and improving cognitive and academic outcomes (more specifically, math achievement).

Research has shown that the ability to control the focus of one’s attention (attentional control or cognitive control) is vital to learning and requires top-down cognitive processes to coordinate thoughts and behaviors to achieve a goal [5]. Attentional control theory [6] proposes that anxiety upsets the balance between the top-down (goal-driven) and bottom-up (stimulus-driven) attentional processes such that it is associated with increased activation of the stimulus-driven system (ie, attention to internal and external stimuli) and decreased influence of the goal-directed system (ie, attention to task demands). Furthermore, attentional control theory posits that highly anxious individuals direct their attention toward potentially threatening information (eg, worrisome thoughts) which in turn reduces the ability to perform ongoing tasks. The theory suggests that the cognitive processes or executive functions most affected by anxiety are inhibitory control, which requires inhibiting distractors and withholding a dominant response, shifting, which entails switching between tasks or demands of a task, and updating, which requires monitoring and updating information in working memory; see Miyake et al [7]. For example, an anxious child might direct their attention to emotional thoughts and be unable to inhibit distracting worries such as “this work is too hard” or “I might fail” and have difficulty shifting their focus back to the task at hand, and when new information is presented requiring them to update their working memory, the demands of controlling attention are borne out in poorer task performance.

There is growing empirical support for targeting or training the cognitive processes most vulnerable to anxiety (ie, inhibitory control, shifting, and updating) and reducing emotional symptoms in adults [8-12] and adolescents [13]. These paradigms are known by the umbrella term, cognitive control training (CCT) or if directly targeting the inhibitory control function, inhibitory control training. A small number of CCT studies have been conducted with children. Results from a systematic review [14] highlighted that of the 8 studies that showed promise for CCT to reduce anxiety only 2 were conducted with primary school-aged children (younger than 12 years of age). Bigorra et al [15] used commercial working memory training with children of 7-12 years with attention-deficit or hyperactivity disorder (ADHD) and behavioral difficulties and Shanok et al [16] used inhibitory control training with typically developing children aged 8-12 years. Both studies reported reduced anxiety in combination with improved cognitive performance (working memory and inhibitory control, respectively); however, neither study examined the transfer of these effects to educational outcomes.

Research in math achievement in school students supports the importance of cognitive control on executive functioning. For instance, Bull and Lee [17] suggest that during math problem-solving, inhibitory control is needed to suppress unwanted information or inappropriate strategies or prepotent number representations, shifting is required to switch math operations within and between more complex problems, and updating is vital for holding and monitoring information in working memory. However, age-related variances in the development of executive function need consideration, particularly as math skill requirements change across the school years [17]. Updating is associated with math achievement in preschoolers [18] and inhibitory control and shifting have also been implicated in early math skill variability [19]. In primary school children, nonetheless, inhibitory control has been shown to be vital for math achievement [20]. Given that the development of executive functioning increases rapidly in the early years of schooling [19,21] and attenuates in adolescence [22], it is plausible that targeting inhibitory control in children aged 8-10 years may serve to boost math performance at a critical time point. The focus of this study is to use a CCT paradigm to reduce anxiety, improve inhibitory control and in turn, math achievement.

### This Study

This proof-of-concept study will examine the effect of inhibitory control training in typically developing children aged 8-10 years. We will compare emotional symptoms (indexed using self-reported scales of anxiety and correlates), inhibitory control, shifting and updating skills (assessed using computerized tasks), and math achievement (measured using standardized math tests) of children completing 15 sessions of daily inhibitory control training versus an active control task from pre- to posttraining and at 1- and 3-month follow-ups. The study will be conducted in year 4 and 5 classrooms. This study aims to determine whether 10 minutes of daily inhibitory control training for 3 weeks can reduce anxiety (and correlates) and improve math achievement and whether any changes are maintained 1 and 3 months after training.

## Methods

### Ethical Considerations

The University of Queensland Human Research Ethics Committee provided approval to conduct this research (2023/HE000462). Gatekeeper approval was also received from participating education departments and schools. Parent consent and child assent will be gained prior to participation. The trial was registered on the Open Science Framework (ofs.io/de2qa).

### Study Design

**Figure 1:**
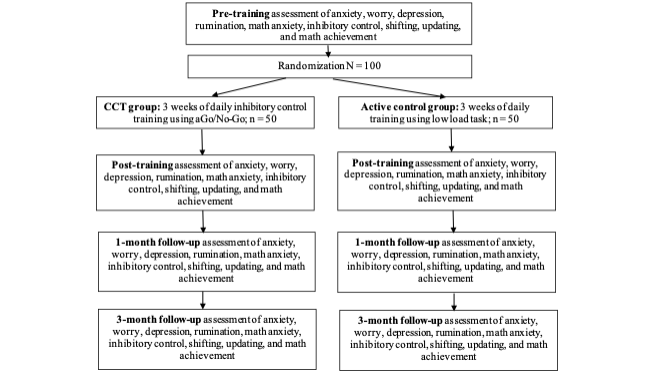
Study flowchart.

We will use a 2 (group: CCT, adaptive Go/No-Go vs active control, low-load task) multiplied by 4 (time: pre- vs posttraining vs 1-month vs 6-month follow-up) randomized design (see Figure 1). Children in both the CCT and active control groups will train for 10 minutes each day for 3 weeks (15 sessions) as part of their daily classroom routine. Changes in the dependent variables (anxiety, worry, depression, rumination, inhibitory control, shifting, updating, and math achievement) will be examined pre- to posttraining, and compared to 1- and 3-month follow-up.

### Participants

A nonselect sample of children aged 8-10 years will be recruited from primary schools. An a priori power analysis revealed that 66 participants are required to detect small effects (*d*=0.20) approaching those reported by previous studies with 80% or greater power on primary outcome measures relative to controls [9,23,24]. To allow for approximately 30% attrition we will recruit 100 children proportionately balanced for sex and age across 8, 9, and 10 years. To incentivize enrollment and study completion, participating children will be compensated using monetary tokens redeemed at the end of the study; Aus $ 2.00 (US $1.31) for the completed questionnaires at each of 4 time points and Aus $ 2.00 (US $1.31) for each training session (~Aus $ 40.00; US $26.14 per participant). Given that teachers are required to supervise and monitor the daily training, they will be incentivized for their class’s participation (minimum 20 students) at the rate of 1 teacher relief day for each data collection time point (pre- and posttraining and 1- and 3-month follow-ups).

Primary and P-12 schools, as well as private, independent, and Catholic schools, will be approached regarding participation in the study. A recruitment package of materials (including a university ethics approval letter, gatekeeper permission request, pamphlet, study protocol, participant information sheets for parents, teachers and children, consent forms, and an information sheet about children’s anxiety and depression) will be sent to the school principal to determine interest in participation. Information sessions will be held face-to-face or using a video communications platform (eg, Zoom) for principals, teachers, and parents, as required.

### Measures and Tasks

#### Anxiety

Anxiety will be measured using the Spence Children’s Anxiety Scale [25], a self-report assessment of specific anxiety symptoms in children aged 8-11 years, categorized as social phobia (6 items), panic or agoraphobia (9 items), generalized anxiety (6 items), obsessive-compulsive (6 items), separation anxiety (6 items), and physical injury fears (5 items). Children respond using a 4-point Likert scale from 0=never to 3=always. Anxiety symptoms will be examined separately (eg, social phobia, and separation anxiety) in addition to the inspection of change in an overall anxiety score calculated by summing the 38 items. Higher scores represent higher symptoms of anxiety.

#### Worry

Worry will be assessed using the Penn State Worry Questionnaire for Children [26], a 14-item questionnaire that measures the tendency to engage in excessive, generalized, and uncontrollable worry in children aged 7-17 years. Items are rated on a 4-point Likert scale from 0=never to 3=always. Total worry scores are calculated (after reversing 3 items) by summing the scores on all items and range from 0 to 42; higher scores represent a greater tendency to worry.

#### Depression

Depression will be indexed by the Low Mood subscale from the Revised Children’s Anxiety and Depression Scale [27], a self-report assessment of symptoms of major depressive disorder in children aged 8-18 years. The low mood subscale includes 10 items and children respond using a 4-point Likert scale from 0=never to 3=always. Total scores range from 0 to 30 with higher scores indicative of lower mood.

#### Rumination

The rumination subscale from the Child Response Style Questionnaire [28] will be used to capture repetitive negative thinking or rumination. Children respond to 13 items on a 4-point Likert scale from 0=almost never to 3=almost always. Total scores range from 0 to 39 with higher scores representing greater rumination.

#### Math Anxiety

The Modified Abbreviated Math Anxiety Scale [29] will be used to assess math anxiety. The 9-item self-report measure comprises 2 subscales, math evaluation anxiety (4 items), for example, thinking about a math test the day before you take it, and math learning anxiety (5 items), for example, starting a new topic in math. Children respond to statements asking how anxious they would feel during certain situations involving math using a 5-point Likert scale from 1=low anxiety to 5=high anxiety.

#### Inhibitory Control

A standard (ie, nonadaptive) Go/No-Go Task [30] will be used to measure inhibitory control. The Go/No-Go Task requires children to view visually presented stimuli and inhibit a dominant response based on instructions, for example, press the spacebar when they see a particular target (go) but do not press the spacebar when they see a different target (no-go). The task captures accuracy and reaction time (RT).

#### Shifting

The Color Shape-Shifting Task [31] will be used to assess shifting performance. In this task, children are presented with some colored shapes and are required to sort the stimuli by shape or color, as fast as they can. Children are given letter cues; for example, *S* for shape and *C* for color before each stimulus appears to indicate the characteristic to focus on. The task captures accuracy and RT.

#### Updating

The *n*-back Task [32] will be used to index updating performance. The task requires children to monitor letters presented in blocks of increasing difficulty (ie, *n*) and indicate when presented with a letter seen on the previous trial (1-back), after 1 intervening trial (2-back), or after 2 intervening trials (3-back). Difficulty increases with the number of intervening trials. Children indicate the same or different with a keyboard button press based on the letter 1-, 2-, or 3-back from this letter. The task captures accuracy and RT.

#### Math Achievement

A total of 3 subscales from the Woodcock-Johnson IV Tests of Achievement [33] will be used to assess math achievement, namely, Applied Problems, Calculation, and Math Fact Fluency. Administration and scoring will be consistent with the authors’ manual. To avoid measurement error due to the practice effects, different test forms will be used at each data collection point.

#### Training Groups

We will randomly assign participants to 1 of the 2 training groups. Groups comprise an experimental CCT group that will train using an adaptive Go/No-Go Task (aGo/No-Go) targeting inhibitory control, and an active control group that will train using a low-load task. The active control task does not require the same cognitive load as the aGO/No-Go task; thus, we do not expect it to produce emotional or cognitive changes. Children in both groups will undertake 10 minutes of training per day for 3 weeks, allowing 15 sessions to be completed.

#### Inhibitory Control Training

The aGo/No-Go task requires participants to focus on desired cues related to a continuous stream of blended stimuli and press the spacebar when the desired cue appears and withhold a response when it does not appear (as per Go/No-Go). The adaptive nature of the task adjusts to the child’s performance such that if the child is doing well, the task reduces the time for responding, whereas if the child is having difficulty the task allows more time for responding, thus positively reinforcing success.

#### Active Control Training

The low-load task presents children with a continuous stream of blended stimuli and requires them to identify target items.

### Equipment and Procedure

All assessments and training will be completed on iPads (9th generation, iOS 16, 64GB; Apple Inc) specific to this project. Self-report symptom scales will be hosted on a computer-based survey platform (Qualtrics) such that the participating children can complete them under the supervision of their classroom teacher, during class time. Inquisit cognitive tasks will be deployed (retrieved from Millisecond Test Library). Math achievement tests will be administered 1:1 in a quiet room by the research team. Daily training of the aGo/No-Go and 1-back will be conducted using a computer-based experiment builder (Gorilla Experiment Builder) and include some minor gamification features to improve motivation and engagement. Training will be completed under the supervision of the classroom teacher.

### Data Analytic Plan

Mixed between-within ANOVA will be used with Group (CCT, aGo/No-Go vs active control; low-load task) as the between-subjects factor and time (pre- vs posttraining vs 1- vs 3-month follow-up) as the within-subjects factor. Separate tests will be conducted with the emotional symptom scores, cognitive measures (ie, accuracy and RT), and math achievement scores as dependent variables. Intention-to-treat analyses will be used to account for missing data, as appropriate. Descriptive and inferential statistics will be performed using SPSS (IBM Corp). We will apply Bonferroni corrections to follow-up tests to control for type 1 error.

## Results

Recruitment and testing opened in September 2023 and will continue for 12 months. We will begin analyzing our data on the completion of the data collection, and the publication of results is expected by the end of 2024.

## Discussion

This study will examine the effect of inhibitory control training on anxiety and math achievement in children aged 8-10 years, relative to an active control condition. We predict children in the CCT group will report reduced emotional symptoms (anxiety, worry, depression, and rumination) and demonstrate improved inhibitory control, shifting, updating, and math achievement when comparing pre- to posttraining, 1-, and 3-month follow-up, relative to controls. Results will inform whether inhibitory control training affords promise as an intervention for anxiety in this age group and advance knowledge of factors affecting math achievement. These findings would provide a foundation for the development of a training app that has the capacity to reach those not well-served, be delivered on large scale for low cost, and afford the possibility of administration across a wide range of settings [4].

This research has several limitations worth noting. We realize using a small sample of children aged 8-10 years means we may not be able to generalize our findings to a larger population or a broader age group. However, pending our results, we aim to replicate our work using a larger cohort and a randomized control design. There are constructs outside the scope of this project. For example, our design will be unable to determine whether inhibitory control training affects other educational outcomes, especially language and reading, which are linked to math achievement. With these potential shortcomings in mind, we hope this work becomes the catalyst for other research using CCT paradigms with children.

## Appendix

Multimedia Appendix 1 Peer reviews.

## Abbreviations

ADHD: attention-deficit/hyperactivity disorder
aGo/No-Go: adaptive Go/No-Go
CCT: cognitive control training
RT: reaction time

## Data Availability

Data generated during this study are available from the corresponding author upon reasonable request.
