# Supplementary material for: Inhibitory Control Training for Anxiety and Math Achievement in Primary School Children: Protocol for a Proof-of-Concept Study
Source: JMIR Res Protoc. 2024 Mar 13;13:e52929. doi: 10.2196/52929 (PMC10973952; doi:10.2196/52929)
Supplement: Multimedia Appendix 1 [file resprot_v13i1e52929_app1.pdf]

#10041299, Edwards/University of Queensland

Review Form Rating Scale:
4-Strongly Agree
3-Agree
2-Disagree
1-Strongly Disagree

Reviewer 1 Comments:

<u>Significance of Research Questions</u>

3-Agree - The proposed research is focused on topics or issues related to learning or education, broadly conceived.
3-Agree - The topics or issues are critical to the field.
3-Agree - The research questions and/or direction of inquiry are clear and compelling.

Significance Comments:
I think this project does related to learning and I do think that mental health and anxiety in children is important. I applaud research that looks to support children in school with anxiety. I would however like to see a bit more explicit connection to the perceived general anxiety and math, rather than math anxiety. I think the PI could have been more explicit with the connection and also make it clear that it is generalized anxiety and not math-specific anxiety. I would also have appreciated perhaps an overarching research question, although I will say they are clear. I hope to learn more about why the limit to 8-10 year olds and if this age group makes the most sense with the anxiety that is evident and also the application of paradigms with adult sto children.

<u>Connection to Research and Theory</u>

4-Strongly Agree - Relevant research literature is used effectively in the proposal to justify the proposed work.
4-Strongly Agree - Theory is used to explain how the proposed research will contribute to or challenge current understandings of education.

Connection to Research and Theory Comments:
I found the PI's literature review/rationale to be very compelling. The PI provided a clear case for by 8-10 years that was build on current situations as well as literature. The PI also makes the connection to math more explicit. Both were questions from the summary that the PI addressed in a clear and cohesive manner. The PI provides an ample amount of theory and prior research, given the space allocated for the overall proposal. The PI clearly has done related work, which I appreciate the mention in the literature review. I also felt that the theory was well connected and not just an add-on.

<u>Research Design</u>

3-Agree - Context or research sites, as well as potential study participants including access and sampling approaches (when appropriate), are sufficiently well-described.
4-Strongly Agree - Sources of data and/or collection plans are clearly identified and well justified.
4-Strongly Agree - Analytic methods are clearly stated and it is evident how the data will be used to answer the proposed research questions and/or support the direction of inquiry.

Design Comments:
I very much appreciate the PI's figure in the research design; I found it helpful and beneficial beyond just additional text. The same is true for Table 1. I found both ways beneficial ways to communicate content. The PI conducted an a priori power analysis, which I significantly appreciate. The PI even accounted for attrition. I found the that I could easily follow along with the methods the PI provided. if I had two critique, one would be that the data analytic plan was a little light but the PI did specify what were the analyses planned and I understand space is a limitation. The second might be more attention to how selecting the 5 primary schools and if the PI considered other demographics than sex and age in their participant selection (e.g., SES, race/ethnicity).

Budget and Timeline Comments:
I only question the Open Science charge, but recognize that is occurring more and more.

Potential of the Research Team Comments:
I have no concerns.

Recommendation Comments:
I found the proposal well written and well conceptualized. I felt the PI described the problem, provided relevant literature and theory, and proposed a research plan to study the identified challenge. I found this easy to read and gain a solid sense of what would occur. I identified a few minor limitations of the proposal, but which are without and space is a limitation:
• The data analytic plan was a little light but the PI did specify what were the analyses planned and I understand space is a limitation.
• Greater attention to how selecting the 5 primary schools and if the PI considered other demographics than sex and age in their participant selection (e.g., SES, race/ethnicity).

Reviewer 2 Comments:

Significance of Research Questions

4-Strongly Agree - The proposed research is focused on topics or issues related to learning or education, broadly conceived.
4-Strongly Agree - The topics or issues are critical to the field.
4-Strongly Agree - The research questions and/or direction of inquiry are clear and compelling.

Significance Comments:

The authors situate their proposal in the COVID-19 pandemic, citing statistics about the prevalence of anxiety in children during the pandemic. The propose an intervention meant to improve mathematics achievement and reduce anxiety by increasing attention (or more directly, increasing inhibitory control) in children. There are clearly achievement-related issue arising from the COVID-19 pandemic as well as mental health correlates in need of urgent research, which this study aims to do. So, the topic is clearly related to education and the issue critical to the field. The basic question, does an intervention improve outcomes, is clear.

Connection to Research and Theory

2-Disagree - Relevant research literature is used effectively in the proposal to justify the proposed work.
2-Disagree - Theory is used to explain how the proposed research will contribute to or challenge current understandings of education.

Connection to Research and Theory Comments:
The connection to the literature feels confusing. The authors cite prior research finding that anxiety can inhibit attentional processes, which in turn harm achievement. However, their apparent theory of change is that intervening on attentional processes might reduce anxiety, thus improving achievement. The causality seems flipped. If anxiety causes inattention, why would increased attention reduce anxiety? It really reads like the authors are trying to add a mental health overlay to a very traditional attention training kind of an approach, but it doesn't really fit with the literature they've cited.

Research Design

2-Disagree - Context or research sites, as well as potential study participants including access and sampling approaches (when appropriate), are sufficiently well-described.
2-Disagree - Sources of data and/or collection plans are clearly identified and well justified.
4-Strongly Agree - Analytic methods are clearly stated and it is evident how the data will be used to answer the proposed research questions and/or support the direction of inquiry.

Design Comments:
The design and analysis are basically fine. Randomizing students to treatment groups is a good practice, and the use of repeated measures resulting in a mixed-between design is appropriate. I wish there were more offered here about the participants and anticipated sample characteristics. For example, have the authors attended to issues of rural/urban location, racial composition and diversity, gender, language, ability/disability, etc.? I was a bit surprised by the selection of Woodcock-Johnson tests for mathematics achievement, but I think it's probably a defensible selection. Mainly, the lack of information about sampling is the major shortfall in this proposal in terms of methodological approach.

Budget and Timeline Comments:
My main comment on the budget is that $2 per session is quite a low participant compensation ($40 total), especially given the time commitment required.

Potential of the Research Team Comments:
Appear capable of the proposed scope of work.

Recommendation Comments:
There are two major issues that I see as issues the authors can address related to this proposal: First, the implied theory of change does not align with the literature as cited, making the mental health correlates feel like they're sort of tacked on to a very traditional project. I don't mean to imply the authors actually did tack them on that way, but it can read that way as presented. The causality or theoretical model just doesn't add up as presented. Secondly, there is very little information on sampling strategy or research sites, which makes it difficult to meaningfully evaluate the representativeness, equity, or diversity of the sample, meaning I cannot comment meaningfully on the appropriateness of the sampling strategy.
